# Protection of COVID-19 Vaccination Against Hospitalization During the Era of Omicron BA.4 and BA.5 Predominance: A Nationwide Case–Control Study Based on the French National Health Data System

**DOI:** 10.1093/ofid/ofad460

**Published:** 2023-09-08

**Authors:** Laura Semenzato, Jérémie Botton, Stéphane Le Vu, Marie-Joëlle Jabagi, François Cuenot, Jérôme Drouin, Rosemary Dray-Spira, Alain Weill, Mahmoud Zureik

**Affiliations:** EPI-PHARE Scientific Interest Group in Epidemiology of Health Products from the French National Agency for the Safety of Medicines and Health Products and the French National Health Insurance, Saint-Denis, France; EPI-PHARE Scientific Interest Group in Epidemiology of Health Products from the French National Agency for the Safety of Medicines and Health Products and the French National Health Insurance, Saint-Denis, France; Faculty of Pharmacy, Paris-Saclay University, Orsay, France; EPI-PHARE Scientific Interest Group in Epidemiology of Health Products from the French National Agency for the Safety of Medicines and Health Products and the French National Health Insurance, Saint-Denis, France; EPI-PHARE Scientific Interest Group in Epidemiology of Health Products from the French National Agency for the Safety of Medicines and Health Products and the French National Health Insurance, Saint-Denis, France; EPI-PHARE Scientific Interest Group in Epidemiology of Health Products from the French National Agency for the Safety of Medicines and Health Products and the French National Health Insurance, Saint-Denis, France; EPI-PHARE Scientific Interest Group in Epidemiology of Health Products from the French National Agency for the Safety of Medicines and Health Products and the French National Health Insurance, Saint-Denis, France; EPI-PHARE Scientific Interest Group in Epidemiology of Health Products from the French National Agency for the Safety of Medicines and Health Products and the French National Health Insurance, Saint-Denis, France; EPI-PHARE Scientific Interest Group in Epidemiology of Health Products from the French National Agency for the Safety of Medicines and Health Products and the French National Health Insurance, Saint-Denis, France; EPI-PHARE Scientific Interest Group in Epidemiology of Health Products from the French National Agency for the Safety of Medicines and Health Products and the French National Health Insurance, Saint-Denis, France; Paris-Saclay University, UVSQ, Paris-Sud University, Inserm, Anti-infective Evasion and Pharmacoepidemiology Unit/Team, CESP, Montigny le Bretonneux, France

**Keywords:** COVID-19, SARS-CoV-2 Omicron variants, SNDS, hospitalization, vaccination

## Abstract

**Background:**

Knowing the duration of effectiveness of coronavirus disease 2019 (COVID-19) booster doses is essential to providing decision-makers with scientific arguments about the frequency of subsequent injections. We estimated the level of protection against COVID-19-related hospitalizations (Omicron BA.4-BA.5) over time after vaccination, accounting for breakthrough infections.

**Methods:**

In this nationwide case–control study, all cases of hospitalizations for COVID-19 identified in the comprehensive French National Health Data System between June 1, 2022, and October 15, 2022, were matched with up to 10 controls by year of birth, sex, department, and an individual COVID-19 hospitalization risk score. Conditional logistic regressions were used to estimate the level of protection against COVID-19-related hospitalizations conferred by primary and booster vaccination, accounting for history of severe acute respiratory syndrome coronavirus 2 (SARS-CoV-2) infection.

**Results:**

A total of 38 839 cases were matched to 377 653 controls; 19.2% and 9.9% were unvaccinated, respectively, while 68.2% and 77.7% had received ≥1 booster dose. Protection provided by primary vaccination reached 45% (95% CI, 42%–47%). The incremental effectiveness of booster doses ranged from 69% (95% CI, 67%–71%; ≤2 months) to 22% (95% CI, 19%–25%; ≥6 months). Specifically, the second booster provided an additional protection compared with the first ranging from 61% (95% CI, 59%–64%; ≤2 months) to 7% (95% CI, 2%–13%; ≥4 months). Previous SARS-CoV-2 infection conferred a strong, long-lasting protection (51% ≥20 months). There was no incremental effectiveness of a second booster among individuals infected since the first booster.

**Conclusions:**

In the era of Omicron BA.4 and BA.5 predominance, primary vaccination still conferred protection against COVID-19 hospitalization, while booster doses provided an additional time-limited protection. The second booster had no additional protection in case of infection since the first booster.

Mass vaccination resulted in a significant decrease in hospitalizations and mortality from coronavirus disease 2019 (COVID-19) [1–4]. However, an upsurge of severe COVID-19 has been observed in vaccinated populations and might be linked to a decline in vaccine effectiveness over time [5] and/or the emergence of new variants. Since mid-June 2022, the Omicron BA.4 and BA.5 subvariants have become the predominant strains circulating in France [6]. This prompted authorities to recommend a first booster dose with the mRNA vaccines, which was rapidly expanded to all adults on November 27, 2021. A second booster dose was recommended in March 2022 [7], initially limited to the oldest individuals and then offered in July 2022 to individuals at risk for a severe form of the disease [8, 9]. In June 2022, ∼60% of the French population had received at least 1 booster dose, with little evolution since [10].

While a large part of the world's population has now acquired some form of immunity to severe acute respiratory syndrome coronavirus 2 (SARS-CoV-2) [11], either through previous infection or vaccination, the question of the durability of the protection over time remains crucial to mitigate the impact of the disease. In a recent systematic review [12], protection against severe disease was maintained at a relatively high level up to 1 year after infection. In a previous study [13], we estimated that the effectiveness of the first booster against hospitalization for COVID-19 reached 83% and then decreased to 78% after 4 months. However, this study did not consider the second booster, did not cover the currently circulating Omicron BA.4 and BA.5 subvariants, and did not consider the interplay between prior SARS-CoV-2 infection and vaccination in maintaining protection.

Here, our objective was to estimate the level of protection against hospitalization for COVID-19 (Omicron BA.4 and BA.5) over time after vaccination, accounting for breakthrough infections.

## METHODS

### Data Sources

This matched case–control study used data from the national COVID-19 vaccination database (VAC-SI: vaccine products and injection dates), coupled with the SARS-CoV-2 diagnosis testing database (SI-DEP: information on positive tests by reverse transcription polymerase chain reaction or antigenic tests) and with the National Health Data System (SNDS), which covers the entire French population (67 million residents). Each person is anonymously identified by a unique, lifelong number. Since 2006, the SNDS has recorded all reimbursement data for outpatient care, including drugs, imaging, and laboratory tests; inpatient care (including diagnoses and procedures performed) from the national hospital discharge database (Programme de Médicalisation des Systèmes d’Information [PMSI]); and health expenditure for patients with long-term diseases, such as cancer and diabetes, which are fully reimbursed in France. Information on hospital stays is collected monthly in the PMSI, and since July 2020, all COVID-19-related hospital stays have been reported through a fast-track procedure. Our study was based on data from this fast-track PMSI database available as of December 9, 2022, with data completeness of about 40% for October. The SNDS has been extensively used to conduct real-life pharmacoepidemiological studies, including on the COVID-19 pandemic [1, 2, 14–16].

### End Point, Design, and Study Population

The primary outcome was hospital admissions with a principal or related diagnosis of COVID-19 [1]. Cases of COVID-19 diagnosis during a hospitalization for another reason (associated diagnosis) were not included. In this case–control study, cases were all individuals aged 12 years or over admitted to the hospital for COVID-19 between June 1, 2022, and October 15, 2022. If several hospitalizations occurred during this period, the first was considered. It should be noted that only very rare cases of re-hospitalization occurred during this period. Because our study related to the Omicron BA.4 and BA.5 variants, we included patients hospitalized for COVID-19 with no history of hospitalization for COVID-19 in the past 3 months [17]. To identify controls at similar risk for severe COVID-19, we constructed an individual risk score [18] for COVID-19 hospitalization based on previous reported associations [19] from a Cox proportional hazards model between the risk of hospitalization for COVID-19 and ∼50 medical conditions plus sociodemographic characteristics, measured in 28 million individuals with a complete primary vaccination in 2021 (details of selected pathologies are given in Table 1). The risk score was calculated as the sum of each identified risk factor (pathologies and sociodemographic characteristics) weighted by the association coefficient obtained by the multivariable Cox model. This score was calculated for the nearly 60 million French residents aged 12 years and older, not deceased as of June 1, 2022, and with at least 1 health care expenditure in 2021. It was discretized into 20 classes based on the quantiles of distribution among the cases included. Each case of hospitalization was then matched on the date of hospital admission (index date) by year of birth, sex, department, and COVID-19 hospitalization risk score class with up to 10 controls without a history of hospitalization for COVID-19 in the past 3 months at the index date. For all matched individuals (each case and its controls), the index date corresponds to the date of hospital admission of the case.

**Table 1.** Characteristics of Hospitalized Cases and Their Controls

	Case	%	Control	%
Sociodemographic characteristics	38 839		377 653	
Age, mean (SD), y	75.0 (17.8)		75.2 (17.6)	
Age				
12–34 y	2014	5.2	19 380	5.1
35–44 y	1121	2.9	10 415	2.8
45–54 y	1817	4.7	16 959	4.5
55–64 y	3237	8.3	30 619	8.1
65–69 y	2458	6.3	23 518	6.2
70–74 y	3895	10.0	38 172	10.1
75–79 y	4643	12.0	45 610	12.1
80–84 y	5533	14.3	54 667	14.5
85–89 y	6925	17.8	68 830	18.2
90+ y	7196	18.5	69 483	18.4
Sex				
Female	19 131	49.3	185 723	49.2
Male	19 708	50.7	191 930	50.8
Region				
Auvergne-Rhône Alpes	4548	11.7	44 032	11.7
Bourgogne Franche Comté	1821	4.7	17 148	4.5
Bretagne	1982	5.1	19 200	5.1
Centre-Val de Loire	1415	3.6	13 556	3.6
Corse	207	0.5	2013	0.5
DOM	1506	3.9	14 922	4.0
Grand Est	3190	8.2	30 524	8.1
Hauts de France	3730	9.6	36 789	9.7
Ile de France	5625	14.5	55 653	14.7
Normandie	1974	5.1	19 206	5.1
Nouvelle Aquitaine	3575	9.2	34 220	9.1
Occitanie	3514	9.1	33 757	8.9
Pays de Loire	1849	4.8	17 889	4.7
Provence Alpes Cote	3903	10.1	38 744	10.3
Social deprivation index (quintiles)				
1 (the least deprivation)	6079	15.7	66 040	17.5
2	6338	16.3	62 275	16.5
3	7605	19.6	67 388	17.8
4	7724	19.9	69 640	18.4
5 (the most deprivation)	8802	22.7	90 021	23.8
Unknown	2291	5.9	22 289	5.9
Lifestyle habits				
Smoking	3019	7.8	20 052	5.3
Alcoholism	924	2.4	7058	1.9
Opioid addiction	160	0.4	510	0.1
Immunosuppressive treatments				
Immunosuppressant	1612	4.2	15 741	4.2
Oral corticosteroids	2326	6.0	18 752	5.0
Comorbidities				
Cardiometabolic diseases				
Diabetes non-insulin-treated	6124	15.8	66 407	17.6
Diabetes insulin-treated	3115	8.0	27 347	7.2
Obesity	334	0.9	4196	1.1
Dyslipidemia and lipid-lowering treatments	13 337	34.3	126 749	33.6
Hereditary metabolic diseases or amyloidosis	200	0.5	1774	0.5
Hypertension	25 425	65.5	246 318	65.2
Coronary diseases	6132	15.8	57 601	15.3
Obliterating arterial disease of the lower limb	2429	6.3	19 984	5.3
Cardiac rhythm or conduction disturbances	9511	24.5	88 923	23.6
Heart failure	4337	11.2	37 057	9.8
Valvular diseases	2737	7.1	24 251	6.4
Stroke	3647	9.4	27 350	7.2
Respiratory diseases				
Chronic respiratory diseases (excluding cystic fibrosis)	7542	19.4	70 297	18.6
Cystic fibrosis	34	0.1	113	0.0
Pulmonary embolism	643	1.7	5103	1.4
Cancer				
Female breast cancer (active)	297	0.8	3803	1.0
Female breast cancer (under surveillance)	652	1.7	7225	1.9
Colorectal cancer (active)	316	0.8	3574	1.0
Colorectal cancer (under surveillance)	582	1.5	6548	1.7
Lung cancer (active)	356	0.9	3088	0.8
Lung cancer (under surveillance)	208	0.5	2043	0.5
Prostate cancer (active)	541	1.4	4968	1.3
Prostate cancer (under surveillance)	865	2.2	8733	2.3
Other cancers (active)	2592	6.7	28 534	7.6
Other cancers (under surveillance)	2241	5.8	21 564	5.7
Inflammatory and skin diseases				
Chronic inflammatory bowel diseases	296	0.8	2709	0.7
Rheumatoid arthritis and related diseases	713	1.8	8029	2.1
Ankylosing spondylitis and related diseases	281	0.7	3482	0.9
Psoriasis	598	1.5	5629	1.5
Psychological and neurodegenerative diseases				
Neurotic and mood disorders, use of antidepressant treatments	8927	23.0	76 868	20.4
Psychotics disorders, use of neuroleptics treatments	2388	6.2	20 801	5.5
Psychiatric disorders starting in childhood	53	0.1	646	0.2
Down syndrome	47	0.1	367	0.1
Epilepsy	833	2.1	5593	1.5
Multiple sclerosis	348	0.9	1937	0.5
Paraplegia	383	1.0	1882	0.5
Myopathy or myasthenia gravis	161	0.4	1056	0.3
Parkinson disease	1539	4.0	9296	2.5
Dementias (including Alzheimer's disease)	3718	9.6	31 573	8.4
Mental impairment	208	0.5	2050	0.5
Other pathologies				
Hemophilia or severe hemostasis disorders	80	0.2	933	0.3
HIV infection	113	0.3	907	0.2
Liver diseases	971	2.5	8555	2.3
Chronic dialysis	468	1.2	3771	1.0
Renal transplant	557	1.4	3040	0.8
Cardiac transplant	29	0.1	80	0.0
Liver transplant	32	0.1	219	0.1
Lung transplant	52	0.1	107	0.0

### Exposure

We defined vaccination status at the index date by identifying the date and number of injections administered. As the effectiveness of vaccines requires a delay from injection, we only considered administration of injections at least 14 days before the index date. Injections administered within 14 days before the index date were not counted and were therefore considered not administered, as they were not effective yet. The number of doses received and the exposure windows, depending on the interval between the last dosage and the index date, were used to establish vaccination status: 14 days–2 months; 2–4 months; 4–6 months; 6–9 months; ≥9 months. We specifically estimated the effectiveness of the first and second booster doses (the third booster dose affecting only 0.2% of our study population) overall and according to the time since last dose: 14 days–2 months; 2–4 months; ≥4 months. More detailed information on the type of COVID-19 vaccines used in France, as well as on the circulation of variants, is available in the eMethod section in the Supplementary Data.

### Sociodemographic Characteristics and Chronic Diseases

Sociodemographic variables included age, gender, and region of residence. Age was divided into subgroups of 12–34-year-olds, 35–44-year-olds, 45–54-year-olds, and 5-year age groups for those 55 and older. We considered the social deprivation index as an estimation of socioeconomic status. This indicator, at the level of city of residence, has been extensively used and is based on the median household income, percentage of high school graduates in the population over the age of 15, percentage of manual workers in the labor force, and unemployment rate [20].

We defined comorbidities using the Cartographie des Pathologies et des Dépenses, a tool developed from the SNDS and PMSI databases that allows the identification of diseases in a given year through medical algorithms [21] based on the reasons for hospitalization, LTD diagnoses, and/or reimbursement of specific treatments in the previous 4 years. This mapping of diseases and expenditures allowed the identification of patients presenting with 42 of these comorbidities in 2021 (cardiometabolic, respiratory, inflammatory, neurodegenerative diseases, cancer, mental and behavioral disorders, etc.) and was completed by the identification of obese patients, people with Down syndrome, psoriasis, heart, lung, or liver transplant recipients, smokers, alcohol or opioid use disorders, and patients treated with immunosuppressants or oral corticosteroids [19].

History of SARS-CoV-2 infection was identified from positive polymerase chain reaction and antigenic tests and hospitalizations for COVID-19 (excluding, for cases and controls, positive tests <2 months before the index date [22] and hospitalizations <3 months before the index date as possible markers of ongoing infection at the index date).

### Statistical Analysis

Crude associations and multivariable conditional logistic regression models, adjusting for the risk factors that were more common and most strongly associated with odds of hospitalization for severe COVID-19 (ie, deprivation index, tobacco use, immunosuppressive treatment, diabetes, dyslipidemia, hypertensive treatments, chronic respiratory diseases, and other cancers before the index date) were used to estimate protection against hospitalization for COVID-19 after vaccination or prior infection. Multivariable models were also adjusted for the number of doses when estimating protection after a previous infection and for history of SARS-CoV-2 infection when estimating protection after booster vaccination.

Effectiveness was calculated as (1 – odd ratio [OR]) × 100, with the lower and upper limits of the confidence interval calculated as (1 − OR_Upper_) × 100 and (1 − OR_Lower_) × 100, respectively.

The effectiveness of vaccination on the risk of hospitalization for COVID-19 was estimated by considering as reference vaccination status: (i) no vaccination; (ii) a complete primary vaccination (2 doses); (iii) for the second booster dose, a complete primary vaccination followed by a first booster dose.

When estimating the second booster's effectiveness, we also performed stratified analyses according to history of infection after the first booster dose (later referred to as a recent infection). We estimated the effectiveness of hybrid immunity (ie, the combined effectiveness of the second booster dose and a recent infection) relative to those with a first booster dose but without a second booster dose or a recent infection.

### Regulatory Approval and Ethical Aspects

The National Health Data System is a set of strictly anonymous databases comprising all mandatory national health insurance reimbursement data, particularly data derived from the processing of health care claims (electronic or paper claims) and data from health care facilities (PMSI).

EPI-PHARE has direct access to the SNDS from the permanent regulatory access of its constitutive bodies, the French National Agency for the Safety of Medicines (ANSM) and Health Products and the French National Health Insurance (Cnam). This permanent access is given according to the French Decree No. 2016–1871 of December 26, 2016, relating to the processing of personal data called the “National Health Data System” [23] and French law articles Art. R. 1461–13 [24] and 14 [25]. This study was declared before its initiation on the EPI-PHARE registry of studies requiring the SNDS.

## RESULTS

**Figure 1.**
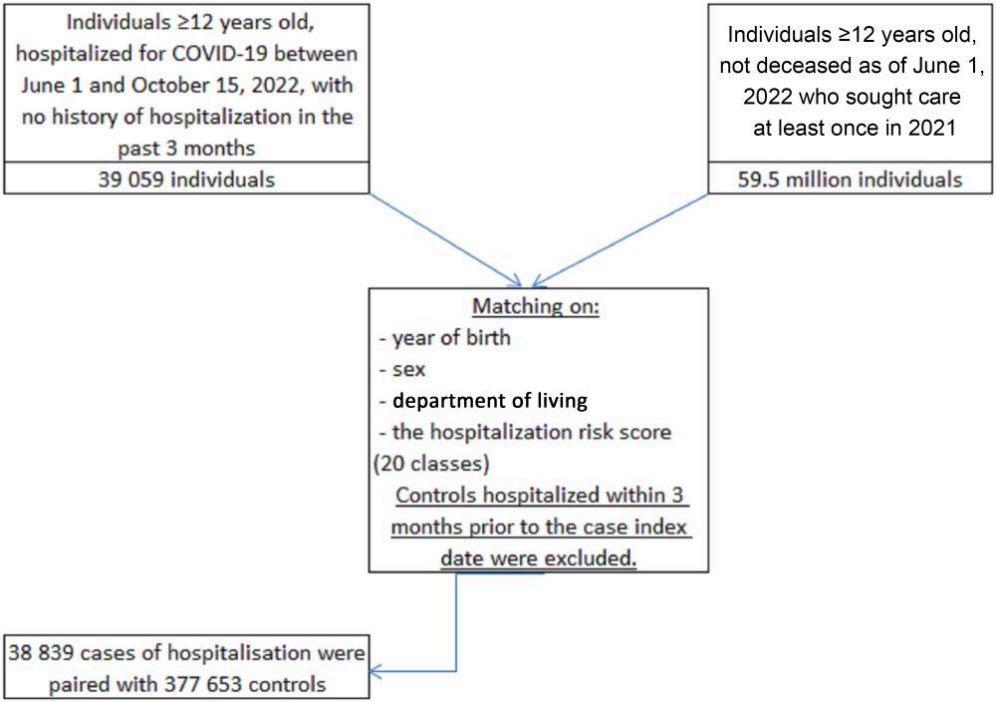
Flow chart.

Between June 1 and 15 October 2022, 39 059 individuals were hospitalized for COVID-19. Among them, 38 839 (>99%) were matched to 377 653 nonhospitalized controls with the same year of birth, sex, area of residence, and COVID-19 hospitalization risk score range (Figure 1). Characteristics of the cases and their controls are presented in Table 1. Cases and controls were, on average, 75 years old; 51% were men and were comparable in medical history, including 24% vs 25% with diabetes, respectively, and 19% with chronic respiratory disease, 65% with hypertension, and 1.5% with lung cancer in both groups.

The proportion of unvaccinated individuals was 19% among cases vs 10% among controls (Table 2). In comparison, 54% and 55%, respectively, had received a first booster dose (average of 7.5 months since the index date), and 14% and 22% had received a second booster dose (average of 3.5 months and 2.8 months since the index date, respectively). The vaccines used as booster doses (first or second booster) were almost exclusively mRNA vaccines (Supplementary Table 1), with mRNA BNT162b2 vaccine (by Pfizer-BioNTech) at >80% and mRNA-1273 vaccine (by Moderna). Otherwise, 9% and 21% had a history of SARS-CoV-2 infection, respectively. Considering unvaccinated individuals as the reference, the level of protection against hospitalization for COVID-19 provided by vaccination reached 45% (95% CI, 42%–47%) for primary vaccination, 56% (95% CI, 55%–57%) for the first booster dose, and 75% (95% CI, 74%–76%) for the second and third booster doses. Protection against hospitalization was the highest in the first 2 months after the last shot (82%; 95% CI, 80%–83%) and then gradually declined to 52% (95% CI, 50%–54%) after 9 months (mean duration, 10.7 months).

**Table 2.** Effectiveness of Vaccine Boosters on the Risk of Hospitalization for COVID-19 During the Omicron Period According to the Number of Doses Received or the Time Since the Last Dose

	Case	%	Control	%	Vaccine Effectiveness	
					Crude Association, %	Multivariable Model,^a^ %
	38 839		377 653			
**No. of doses**						
Unvaccinated	7454	19	37 442	10	1	1
1 dose	713	2	5727	2	39 (34–44)	29 (22–34)
2 doses	4190	11	41 082	11	51 (49–53)	45 (42–47)
3 doses (first booster)	20 921	54	209 232	55	54 (53–56)	56 (55–57)
4 or 5 doses (second and third boosters)	5561	14	84 170	22	72 (71–73)	75 (74–76)
**Time since last dose**						
Unvaccinated	7454	19	37 442	10	1	1
Partially vaccinated (1 dose only or a 2nd dose <14 d)	713	2	5727	2	39 (34–44)	27 (21–33)
14 d to <2 mo	1402	4	32 066	9	81 (80–82)	82 (80–83)
2–<4 mo	2997	8	45 723	12	71 (70–72)	73 (72–74)
4–<6 mo	4705	12	48 644	13	55 (53–57)	57 (56–59)
6–<9 mo	15 493	40	147 726	39	52 (50–53)	53 (52–55)
≥9 mo	6075	16	60 325	16	55 (53–56)	52 (50–54)
**Time since the last booster dose**						
Complete primary vaccination (2 doses)	4181	14	30 890	12	1	1
Booster dose	26 466	86	237 079	89	15 (12–18)	32 (30–35)
Complete primary vaccination (2 doses)	4181	14	30 890	12	1	1
Booster dose 14 d–<2 mo	1363	4	26 045	10	61 (59–64)	69 (67–71)
Booster dose 2–<4 mo	2892	9	36 899	14	42 (39–45)	55 (53–58)
Booster dose 4–<6 mo	4132	14	33 939	13	9 (5–13)	30 (26–33)
Booster dose ≥6 mo	18 079	59	140 196	52	3 (0–7)	22 (19–25)

Abbreviations: COVID-19, coronavirus disease 2019; SARS-CoV-2, severe acute respiratory syndrome coronavirus 2.

a Adjustment for social deprivation index, tobacco dependence, use of immunosuppressants or oral corticosteroids, history of diabetes, dyslipidemia, hypertension, chronic respiratory disease, other cancers, and history of SARS-CoV-2 infection (occurring before the index date).

After multivariable adjustment including the number of vaccine doses, history of SARS-CoV-2 infection conferred a protection against hospitalization for COVID-19 that varied according to the SARS-CoV-2 variant causing the infection and the duration since infection. This protection ranged from 68% (95% CI, 65%–70%) to 78% (95% CI, 76%–80%) for infections caused by Omicron sublineages that occurred within the 6 preceding months, and from 51% (95% CI, 46%–55%) to 60% (95% CI, 51%–67%) for infections caused by non-Omicron sublineages that occurred up to 20 months earlier (Table 3).

**Table 3.** Association Between History of SARS-CoV-2 Infection and Hospitalization for COVID-19

	Case	%	Control	%	Crude Association, %	Multivariable Model,^a^ %
**History of the last SARS-CoV-2 infection**						
No previous history	35 427	91.2	299 866	79.4	1	1
Omicron sublineages	1727	4.4	50 819	13.5	73 (71–74)	74 (73–76)
Non-Omicron sublineages	1685	4.3	26 968	7.1	52 (49–54)	60 (58–62)
**Time since the last SARS-CoV-2 infection**						
No previous history	35 427	91.2	299 866	79.4	1	1
Omicron sublineages						
SARS-CoV-2 infection: 2–<4 mo	476	1.2	16 822	4.5	77 (75–79)	78 (76–80)
SARS-CoV-2 infection: 4–<6 mo	692	1.8	21 579	5.7	74 (72–76)	76 (74–78)
SARS-CoV-2 infection: ≥6 mo	559	1.4	12 418	3.3	64 (61–67)	68 (65–70)
Non-Omicron sublineages						
SARS-CoV-2 infection: 6–<8 mo	113	0.3	1866	0.5	52 (41–60)	60 (51–67)
SARS-CoV-2 infection: 8–<10 mo	102	0.3	1703	0.5	51 (40–60)	60 (51–67)
SARS-CoV-2 infection: 10–<12 mo	108	0.3	1928	0.5	55 (45–63)	61 (53–68)
SARS-CoV-2 infection: 12–<14 mo	108	0.3	1414	0.4	39 (26–50)	47 (36–57)
SARS-CoV-2 infection: 14–<16 mo	231	0.6	3588	1.0	48 (40–54)	56 (50–62)
SARS-CoV-2 infection: 16–<18 mo	262	0.7	4381	1.2	52 (45–57)	58 (53–63)
SARS-CoV-2 infection: 18–<20 mo	277	0.7	4931	1.3	54 (48–59)	59 (54–64)
SARS-CoV-2 infection: ≥20 mo	484	1.2	7157	1.9	45 (39–50)	51 (46–55)

Abbreviations: COVID-19, coronavirus disease 2019; SARS-CoV-2, severe acute respiratory syndrome coronavirus 2.

a Adjustment for the number of vaccine doses, social deprivation index, tobacco dependence, use of immunosuppressants or oral corticosteroids, history of diabetes, dyslipidemia, hypertension, chronic respiratory disease, and other cancers.

Booster doses conferred an estimated 32% (95% CI, 30%–35%) protection against the risk of hospitalization for COVID-19 compared with primary vaccination (Table 2). This protection ranged from 69% (95% CI, 67%–71%) in the first 2 months after injection to 22% (95% CI, 19%–25%) beyond 6 months. Results did not differ according to age group or gender (Supplementary Table 2). A sensitivity analysis of cases hospitalized for at least 2 days (71% of cases) and their controls (an indicator described as a severity criterion in World Health Organization [WHO] guidelines [26]) yielded similar results (Supplementary Table 3).

The specific incremental effectiveness of the second booster dose compared with the first booster dose, estimated by restricting the analysis to case–control pairs with a first or a second booster dose, that is, 26 332 cases and 206 125 controls (Supplementary Table 4), was 44% (95% CI, 42%–46%) (Table 4). It was 61% (95% CI, 59%–64%) in the first 2 months, 45% (95% CI, 43%–48%) between 2 and 4 months, and 7% (95% CI, 2%–13%) beyond. In this population, 11% had a history of SARS-CoV-2 infection after the first booster dose (3.6% of cases and 12% of controls). The effectiveness of the second booster dose dropped to 2% (95% CI, −18% to 19%) in those with a recent history of infection (Table 4). The effectiveness of hybrid immunity was estimated at 82% (95% CI, 78%–84%) compared with those who did not receive a second booster dose or have a recent history of infection (Supplementary Table 5). Note that we also observed a decrease in the incremental effectiveness of the first booster dose compared with complete primary vaccination (Supplementary Tables 6 and 7).

**Table 4.** Reduced Risk of Hospitalization for COVID-19 in the Omicron Period Associated With the Second Booster Dose Compared With the First Booster Dose, Overall and Taking Into Account History of Infection Between the First Booster Dose and the Two Months Before the Index Date

	Case	%	Control	%	Crude Association, %	Multivariable Model,^a^ %
**Overall**						
First booster dose	20 870	79	144 297	70	1	1
Second booster dose	5462	21	61 828	30	39 (37–41)	44 (42–46)
First booster dose	20 870	79	144 297	70	1	1
Second booster dose: 14 d–<2 mo	1207	5	20 625	10	59 (56–61)	61 (59–64)
Second booster dose: 2–<4 mo	2462	9	28 690	14	40 (37–43)	45 (43–48)
Second booster dose: ≥4 mo	1793	7	12 513	6	2 (−4 to 7)	7 (2–13)
**No history of recent infection**						
First booster dose	20 063	79	123 680	68	1	1
Second booster dose	5319	21	57 654	32	43 (41–45)	44 (42–46)
First booster dose	20 063	79	123 680	68	1	1
Second booster: <4 mo	3594	14	46 244	26	52 (50–54)	52 (50–54)
Second booster: ≥4 mo	1725	7	11 410	6	7 (2–12)	9 (4–14)
**History of recent infection**						
First booster dose	807	85	20 617	83	1	1
Second booster dose	143	15	4174	17	12 (−5 to 27)	2 (−18 to 19)

Abbreviations: COVID-19, coronavirus disease 2019; SARS-CoV-2, severe acute respiratory syndrome coronavirus 2.

a Adjustment for social deprivation index, tobacco dependence, use of immunosuppressants or oral corticosteroids, history of diabetes, dyslipidemia, hypertension, chronic respiratory disease, other cancers, and history of SARS-CoV-2 infection (occurring before the first booster dose).

## DISCUSSION

In this matched case–control study including all hospitalizations for COVID-19 in France between June 1, 2022, and October 15, 2022, compared with no vaccination, effectiveness against the risk of hospitalization for COVID-19 was estimated at 45% for complete primary vaccination, 56% for the first booster dose, and 75% for the second booster dose. Effectiveness was the highest in the 2 months after injection (82%), and then decreased to 52% beyond 9 months (mean duration, 10.7 months). Booster doses provided temporarily increased protection compared with primary vaccination, ranging from 69% within the first 2 months to 22% beyond 6 months. Previous infection conferred strong, long-lasting protection against hospitalization for COVID-19, reaching 70%–80% for Omicron sublineage infections and 50%–60% for infections occurring previously. The second booster provided no additional benefit in protection to individuals who became infected after they received a first booster. Effectiveness of hybrid immunity was 82%.

Compared with unvaccinated individuals, the decrease in vaccine effectiveness of the last dose against the risk of hospitalization for COVID-19 (82% within 2 months vs 52% beyond 9 months) was consistent with other studies [27–34]. A US study by Adams et al. [30] during the Omicron period (December 26, 2021, to June 30, 2022) estimated an effectiveness of the first booster dose against the risk of hospitalization of 76% (95% CI, 69%–81%) within the first 4 months vs 39% (95% CI, 22%–53%) beyond that time and an effectiveness of the second booster dose of 62% (95% CI, 35%–78%) in the first 4 months. Another US study by Ferdinands et al. [28] between January 17, 2021, and July 12, 2022, estimated the effectiveness of the first booster dose against the risk of hospitalization at 89% (95% CI, 88%–90%) during the first 2 months and 66% (95% CI, 63%–69%) after 4 months. As mentioned by Carabelli et al. [35], the successive mutations of the different COVID-19 variants only weakly affected the T-cell epitopes and the associated immune response, which would be likely to maintain vaccine effectiveness (50% beyond 6 months in our study). Compared with individuals with a primary vaccination for at least 25 weeks, an English study by Kirsebom et al. [36] estimated the effectiveness of a first or second booster dose against the risk of hospitalization at 14 weeks to be ∼60% against the BA.4 and BA.5 variants and ∼20% after 24 weeks. In our study, the incremental benefit of the second booster over the first against the risk of severe disease was 44% (95% CI, 42%–46%), ranging from 61% (95% CI, 59%–64%) in the first 2 months to 7% (95% CI, 2%–13%) beyond 4 months. These estimates are consistent with the results of 2 studies among older adults: A Canadian study [37] conducted in long-term care facilities among individuals aged 60 years or older estimated the effectiveness of the second booster dose compared with the first at 40% (95% CI, 24%–52%); an Italian study [38] among individuals aged 80 years or older estimated the effectiveness of the second booster dose compared with the first at 43% (95% CI, 31%–55%) at 1 month and 27% (95% CI, 8–43%) at 4 months. If our study was not limited to the elderly population, the mean age of cases and controls in this subpopulation was 77 and 78 years, respectively (Supplementary Table 4). Although some studies have shown high and continuous effectiveness of boosters over time against the risk of severe forms of COVID-19 [39–41], these studies involved fewer events and a younger population.

We observed a benefit of hybrid immunity conferred by both effectiveness of vaccination and SARS-CoV-2 infection, consistent with several studies that found maximum protection over time with hybrid immunity [42–48]. As in a review by Stein et al. [12], protection of prior infection against a subsequent severe COVID-19 infection appears to last up to >10 months for ancestral variants and even up to 20 months in our study, thus being at least as durable as that provided by 2 doses of vaccination. However, SARS-CoV-2 infection is associated with an increased risk of hospitalization for COVID-19, admission to intensive care, and death, but also, a risk of persistent symptoms, long COVID, and short- and medium-term respiratory, cardiovascular, and neurological complications [49–52], as opposed to vaccination. In case of a recent infection conferring a high level of protection, the effectiveness of a second booster dose appeared lower, probably because people who did not receive a second booster dose were already substantially protected from hospitalization, almost as well as those who did receive a second booster. In addition, the relatively lower effectiveness of vaccination on Omicron variants could be explained by the immune evasiveness properties of the Omicron variants [35], while a booster dose would allow a broader immune response [30].

This study has some limitations. Some SARS-CoV-2 infections are not recorded in the testing database. Some individuals do not want to be tested or have not been tested because of asymptomatic infections, and the results of self-tests are not recorded. This may affect (and probably underestimate) our estimate of hybrid immunity, as individuals in the reference exposure group are probably better protected than actually recorded in our data, and at least partly lead to an artificial decrease in vaccine effectiveness [34, 43]. Vaccine effectiveness may also have been underestimated due to incidental findings of SARS-CoV-2 during hospital admission screening, leading to misclassification of the outcome of severe disease due to Omicron. However, we only included hospitalized cases with a principal or related diagnosis of COVID-19 (no associated diagnosis). And a sensitivity analysis of cases hospitalized for at least 2 days (71% of cases) and their controls, an indicator described as a severity criterion in the WHO guidelines [26], produced similar results. Hospitalization data were complete from June through September, but completeness was 40% for October at the time of the analyses. However, the number of controls for whom hospitalization was not reported is small compared with the total number of controls; therefore, the risk of biasing the estimates is likely limited. By design, the selected control individuals had a higher risk of hospitalization than the general population. Although this makes the estimates more reliable, they may not be generalizable to individuals at lower risk. Characterization of the BA.4 and BA.5 sublineages was unavailable individually in the databases and therefore relied on the period of predominance defined from surveillance data [53]: as of the beginning of June 2022, the BA.5 variant represents >40% and the BA.4 variant 5% of variants in circulation. In mid-June, the BA.5 variant became a 60% majority, reaching >85% of variants in circulation from early July and until the end of the inclusion period in mid-October. Bivalent vaccines were unavailable over the study period, so our results are not generalizable to these vaccines. Finally, despite the matching of cases and controls on COVID-19 hospitalization risk scores and a relatively balanced distribution of the different adjustment variables (including a large number of risk factors for severe COVID-19), unmeasured residual confounding factors, such as risk behaviors (eg, noncompliance with barrier measures), may remain.

This study focuses on the near-completeness of hospitalizations between June 1 and October 15, 2022, when the BA.4 and BA.5 sublineages of the Omicron variant prevailed. Its originality lies in the matching with controls at equivalent risk of hospitalization and in considering recent episodes of infection when estimating booster effectiveness.

## CONCLUSIONS

In the era of Omicron BA.4 and BA.5 predominance, primary vaccination still conferred protection against COVID-19 hospitalization, while booster doses provided additional time-limited protection. The second booster did not provide additional protection in case of infection since the first booster. Further work will be needed to measure protection after bivalent vaccines, which became available in France in October 2022.

## Supplementary Material

ofad460_Supplementary_Data

## Supplementary Data

Supplementary materials are available at *Open Forum Infectious Diseases* online. Consisting of data provided by the authors to benefit the reader, the posted materials are not copyedited and are the sole responsibility of the authors, so questions or comments should be addressed to the corresponding author.
