# Supplementary material for: Protection of COVID-19 Vaccination Against Hospitalization During the Era of Omicron BA.4 and BA.5 Predominance: A Nationwide Case–Control Study Based on the French National Health Data System
Source: Open Forum Infect Dis. 2023 Sep 8;10(10):ofad460. doi: 10.1093/ofid/ofad460 (PMC10551849; doi:10.1093/ofid/ofad460)
Supplement: ofad460_Supplementary_Data [file ofad460_supplementary_data.docx]

**eMethods**

The COVID-19 vaccination campaign began in France on December 27, 2020, first with two messenger RNA (mRNA) vaccines, BNT162b2 vaccine (by Pfizer-BioNTech©) and mRNA-1273 vaccine (by Moderna©), then with adenovirus based vaccine ChAdOx1 nCoV-19 (by Oxford-AstraZeneca©) in February 2021 and lastly with Ad26.COV2.S vaccine (Janssen©) in April 2021.

Target populations were different according to vaccines and there was a prioritisation over time depending on vaccines’ availability: vaccines went first to healthcare workers, individuals living in nursing homes, those aged 75 years or older, and those with severe or multiple chronic conditions and were then extended in mid-April to all people aged 55 and over, in mid-May to all adults, and in mid-June to adolescents aged 12 and over.

The original alpha variant of SARS-COV-2 appeared in France in mid-February 2020 and was the cause of 2 epidemic waves, at the beginning and the end of 2020. It predominates until early 2021 followed by the beta and gamma variants (3^rd^ epidemic wave in April 2021), and then the Delta variant (4^th^ epidemic wave in June 2021)^1^. With the decline in vaccine effectiveness over time^2^, the emergence of new variants and a slackening of preventive measures, an upsurge of severe COVID-19 has been observed in vaccinated populations at the end of 2021.

This prompted authorities to recommend a first booster dose with the mRNA vaccines, which was rapidly expanded to all adults from November 27, 2021. A second booster dose became recommended in March 2022^3^, initially limited to the oldest individuals and then offered in July 2022 to individuals at risk for a severe form of the disease^4,5^.

Based on indicators used to track the circulation of SARS-COV-2 variants in France and derived from sequencing results (flash surveys – EMERGEN)^6^, since late 2021, the Omicron variant has predominated, with the emergence of different sublineages over time: BA.1 variant between January and mid-February 2022 (5^th^ epidemic wave between November 2021 and February 2022), BA.2 variant between mid-February and early June 2022 (6^th^ epidemic wave in April 2022), BA.4 and BA.5 variant during the second semester of 2022 (7^th^ and 8^th^ wave). In 2023, recombinant forms of Omicron prevailed.

1. Santé Publique France. Coronavirus : circulation des variants du SARS-CoV-2. https://www.santepubliquefrance.fr/dossiers/coronavirus-covid-19/coronavirus-circulation-des-variants-du-sars-cov-2.

2. Thomas, S. J. & Moreira, E. D. Safety and Efficacy of the BNT162b2 mRNA Covid-19 Vaccine through 6 Months. *n engl j med* 13 (2021).

3. Conseil d’Orientation de la Stratégie Vaccinale. *Addendum du 18 février 2022 à l’avis du 19 janvier 2022 Opportunité de la mise en place d’une seconde dose de rappel vaccinal*.

4. Gouvernement. Les personnes de 60 ans et plus peuvent effectuer une deuxième dose de rappel contre le Covid-19.

5. Avis n°2022.0043/AC/SESPEV du 13 juillet 2022 du Collège de la Haute Autorité de santé relatif à la place d’une dose de rappel additionnelle des vaccins contre la Covid-19 dans la stratégie vaccinale.

6. Santé Publique France. Monitoring of Omicron sub-lineages by sequencing. https://www.santepubliquefrance.fr/dossiers/coronavirus-covid-19/english-coronavirus-key-numbers-for-covid-19-and-its-evolution-in-france-and-across-the-world.

**Table S1:** Type of vaccine administered at last dose.

|  | **1^st^ dose** | | | | **2^nd^ dose** | | | | **Booster doses (i.e. ≥ 3^rd^ dose)** | | | |
| --- | --- | --- | --- | --- | --- | --- | --- | --- | --- | --- | --- | --- |
| **Type of vaccine** | Case | % | Control | % | Case | % | Control | % | Case | % | Control | % |
|  | 713 |  | 5,727 |  | 4,190 |  | 41,082 |  | 26,482 |  | 293,402 |  |
| PFIZER | 449 | 63% | 3,782 | 66% | 3,105 | 74% | 30,862 | 75% | 21,612 | 82% | 242,489 | 83% |
| MODERNA | 120 | 17% | 1,019 | 18% | 975 | 23% | 9,305 | 23% | 4,865 | 18% | 50,854 | 17% |
| JANSSEN | 121 | 17% | 770 | 13% | 3 | 0% | 10 | 0% |  | 0% | 9 | 0% |
| ASTRAZENECA | 20 | 3% | 139 | 2% | 104 | 2% | 868 | 2% | 4 | 0% | 38 | 0% |
| NOVAVAX | 3 | 0% | 17 | 0% | 3 | 0% | 37 | 0% | 1 | 0% | 12 | 0% |

**Table S2:** Effectiveness of any vaccine booster on reducing the risk of hospitalization for COVID-19 in the omicron period by time since the last dose, and stratified by age (a) and sex (b).

a.

|  | **< 55** | | | | | | **55 to 74** | | | | | | **>= 75** | | | | | |
| --- | --- | --- | --- | --- | --- | --- | --- | --- | --- | --- | --- | --- | --- | --- | --- | --- | --- | --- |
|  | **Case** | **%** | **Control** | **%** | **Crude association** | **Multivariable model*** | **Case** | **%** | **Control** | **%** | **Crude association** | **Multivariable model*** | **Case** | **%** | **Control** | **%** | **Crude association** | **Multivariable model*** |
|  | 3,307 |  | 25,231 |  |  |  | 7,631 |  | 65,973 |  |  |  | 19,709 |  | 176,765 |  |  |  |
| **Last dose (2^nd^ dose)** | 957 | 29% | 7,337 | 29% | 1 | 1 | 1,049 | 14% | 8,641 | 13% | 1 | 1 | 2,175 | 11% | 14,912 | 8% | 1 | 1 |
| **Last dose (any booster dose)** |  |  |  |  |  |  |  |  |  |  |  |  |  |  |  |  |  |  |
| 14 days to < 2 months | 30 | 1% | 306 | 1% | 33% (1% ; 55%) | 45% (18% ; 63%) | 305 | 4% | 5,826 | 9% | 57% (51% ; 63%) | 66% (61% ; 70%) | 1,028 | 5% | 19,913 | 11% | 64% (61% ; 67%) | 70% (67% ; 72%) |
| 2 to < 4 months | 86 | 3% | 567 | 2% | -10% (-40% ; 14%) | 2% (-26% ; 23%) | 448 | 6% | 4,474 | 7% | 19% (9% ; 28%) | 37% (28% ; 44%) | 2,358 | 12% | 31,858 | 18% | 49% (46% ; 52%) | 58% (56% ; 61%) |
| 4 to < 6 months | 692 | 21% | 5,584 | 22% | 5% (-6% ; 15%) | 24% (15% ; 32%) | 1,089 | 14% | 8,734 | 13% | -4% (-14% ; 5%) | 16% (8% ; 24%) | 2,351 | 12% | 19,621 | 11% | 16% (11% ; 22%) | 32% (27% ; 36%) |
| >= 6 months | 1,542 | 47% | 11,437 | 45% | -4% (-14% ; 5%) | 12% (3% ; 20%) | 4,740 | 62% | 38,298 | 58% | -2% (-10% ; 5%) | 14% (8% ; 21%) | 11,797 | 60% | 90,461 | 51% | 9% (4% ; 13%) | 20% (16% ; 24%) |

b.

|  | **Men** | | | | | | **Women** | | | | | |
| --- | --- | --- | --- | --- | --- | --- | --- | --- | --- | --- | --- | --- |
|  | **Case** | **%** | **Control** | **%** | **Crude association** | **Multivariable model** | **Case** | **%** | **Control** | **%** | **Crude association** | **Multivariable model*** |
|  | 16,214 |  | 143,228 |  |  |  | 14,433 |  | 124,741 |  |  |  |
| **Last dose (2^nd^ dose)** | 1,952 | 12% | 15,327 | 11% | 1 | 1 | 2,229 | 15% | 15,563 | 12% | 1 | 1 |
| **Last dose (any booster dose)** |  |  |  |  |  |  |  |  |  |  |  |  |
| 14 days to < 2 months | 791 | 5% | 14,170 | 10% | 55% (51% ; 59%) | 63% (60% ; 66%) | 572 | 4% | 11,875 | 10% | 67% (63% ; 70%) | 73% (70% ; 75%) |
| 2 to < 4 months | 1,597 | 10% | 20,078 | 14% | 37% (32% ; 42%) | 50% (46% ; 53%) | 1,295 | 9% | 16,821 | 13% | 47% (43% ; 51%) | 57% (54% ; 61%) |
| 4 to < 6 months | 2,182 | 13% | 17,671 | 12% | 1% (-5% ; 8%) | 20% (15% ; 26%) | 1,950 | 14% | 16,268 | 13% | 16% (10% ; 21%) | 33% (28% ; 37%) |
| >= 6 months | 9,692 | 60% | 75,982 | 53% | -2% (-8% ; 3%) | 12% (7% ; 17%) | 8,387 | 58% | 64,214 | 51% | 8% (3% ; 13%) | 22% (18% ; 26%) |

***** adjustment for social deprivation index, tobacco dependence, use of immunosuppressants or oral corticosteroids, history of diabetes, dyslipidaemia, hypertension, chronic respiratory disease, other cancers, and history of SARS-COV-2 infection (occurring before the index date)

**Table S3**: Effectiveness of any vaccine booster on reducing the risk of hospitalization for COVID-19 in the omicron period by time since the last dose – sensitivity analysis on cases hospitalized for at least 2 days (severity criterion in the WHO guidance) and their controls (c).

|  | **Cases with hospital stay ≥2 days and their controls** | | | | |
| --- | --- | --- | --- | --- | --- |
|  | **Case** | **%** | **Control** | **%** | **Vaccine effectiveness - Multivariable model*** |
|  | 27,731 |  | 269,592 |  |  |
| **Last dose (2^nd^ dose)** | 2,810 | 13% | 20,240 | 10% | 1 |
| **Last dose (any booster dose)** |  |  |  |  |  |
| 14 days to < 2 months | 1,058 | 5% | 20,335 | 10% | 70% (68% ; 72%) |
| 2 to < 4 months | 2,171 | 10% | 28,839 | 15% | 58% (56% ; 61%) |
| 4 to < 6 months | 2,864 | 13% | 23,223 | 12% | 30% (26% ; 34%) |
| >= 6 months | 13,259 | 60% | 102,482 | 53% | 23% (20% ; 27%) |

***** adjustment for social deprivation index, tobacco dependence, use of immunosuppressants or oral corticosteroids, history of diabetes, dyslipidaemia, hypertension, chronic respiratory disease, other cancers, and history of SARS-COV-2 infection (occurring before the index date)

**Table S4:** Characteristics of hospitalized cases and their controls with a first or a second booster dose.

|  |  | **Case** | **%** | **Control** | **%** |
| --- | --- | --- | --- | --- | --- |
| **Sociodemographic characteristics** |  | 26,332 |  | 206,125 |  |
| **Age (year) - mean (std)** |  | 77.05 (15.1) |  | 78.38 (13.6) |  |
| **Age** | 12-34 | 696 | 2,6 | 3,410 | 1,7 |
|  | 35-44 | 511 | 1,9 | 2,724 | 1,3 |
|  | 45-54 | 1,104 | 4,2 | 6,850 | 3,3 |
|  | 55-64 | 2,063 | 7,8 | 14,232 | 6,9 |
|  | 65-69 | 1,661 | 6,3 | 12,469 | 6,1 |
|  | 70-74 | 2,806 | 10,7 | 22,470 | 10,9 |
|  | 75-79 | 3,403 | 12,9 | 28,170 | 13,7 |
|  | 80-84 | 4,126 | 15,7 | 34,665 | 16,8 |
|  | 85-89 | 5,156 | 19,6 | 43,316 | 21,0 |
|  | 90+ | 4,806 | 18,3 | 37,819 | 18,4 |
| **Sex** | Female | 12,149 | 46,1 | 93,233 | 45,2 |
|  | Male | 14,183 | 53,9 | 112,892 | 54,8 |
| **Region** | Auvergne-Rhône Alpes | 3,128 | 11,9 | 24,378 | 11,8 |
|  | Bourgogne Franche Comté | 1,315 | 5,0 | 10,149 | 4,9 |
|  | Bretagne | 1,527 | 5,8 | 12,818 | 6,2 |
|  | Centre-Val de Loire | 1,044 | 4,0 | 8,360 | 4,1 |
|  | Corse | 126 | 0,5 | 921 | 0,5 |
|  | DOM | 416 | 1,6 | 1,871 | 0,9 |
|  | Grand Est | 2,290 | 8,7 | 17,955 | 8,7 |
|  | Hauts de France | 2,620 | 10,0 | 20,823 | 10,1 |
|  | Ile de France | 3,817 | 14,5 | 29,705 | 14,4 |
|  | Normandie | 1,475 | 5,6 | 12,066 | 5,9 |
|  | Nouvelle Aquitaine | 2,538 | 9,6 | 20,290 | 9,8 |
|  | Occitanie | 2,333 | 8,9 | 17,956 | 8,7 |
|  | Pays de Loire | 1,373 | 5,2 | 11,280 | 5,5 |
|  | Provence Alpes Cote | 2,330 | 8,9 | 17,553 | 8,5 |
| **Social deprivation index (quintiles)** | 1 (the least deprivation) | 4,388 | 16,7 | 37,595 | 18,2 |
|  | 2 | 4,498 | 17,1 | 35,039 | 17,0 |
|  | 3 | 5,160 | 19,6 | 37,551 | 18,2 |
|  | 4 | 5,312 | 20,2 | 39,586 | 19,2 |
|  | 5 (the most deprivation) | 5,979 | 22,7 | 50,178 | 24,3 |
|  | Unknown | 995 | 3,8 | 6,176 | 3,0 |
| **Lifestyle habits** |  |  |  |  |  |
| Smoking |  | 2,110 | 8,0 | 11,288 | 5,5 |
| Alcoholism |  | 615 | 2,3 | 3,876 | 1,9 |
| Opioid addiction |  | 76 | 0,3 | 181 | 0,1 |
| **Vaccination schedule at the index date** |  |  |  |  |  |
|  | 3 injections | 20,870 | 79,3 | 144,297 | 70,0 |
|  | 4 injections | 5,462 | 20,7 | 61,828 | 30,0 |
| **History of SARS-COV-2 infection** |  |  |  |  |  |
| Positive PCR and antigenic tests (excluding positive tests less than 2 months before the index date ) | | 1,352 | 5,1 | 32,087 | 15,6 |
| Hospitalisation for COVID-19 (excluding hospitalisations less than 3 months before the index date ) | | 369 | 1,4 | 3,816 | 1,9 |
| **Immunosuppressive treatments** |  |  |  |  |  |
| Immunosuppressant |  | 1,247 | 4,7 | 9,651 | 4,7 |
| Oral corticosteroids |  | 1,785 | 6,8 | 11,764 | 5,7 |
| **Comorbidities** |  |  |  |  |  |
| **Cardiometabolic diseases** |  |  |  |  |  |
| Diabetes non insulino-treated |  | 4,374 | 16,6 | 39,194 | 19,0 |
| Diabetes insulino-treated |  | 2,144 | 8,1 | 15,716 | 7,6 |
| Obesity |  | 229 | 0,9 | 2,192 | 1,1 |
| Dyslipidemia and lipid-lowering treatments |  | 10,280 | 39,0 | 79,501 | 38,6 |
| Hereditary metabolic diseases or amyloidosis |  | 146 | 0,6 | 1,098 | 0,5 |
| Hypertension |  | 18,391 | 69,8 | 147,827 | 71,7 |
| Coronary diseases |  | 4,620 | 17,6 | 36,188 | 17,6 |
| Obliterating arterial disease of the lower limb |  | 1,783 | 6,8 | 12,292 | 6,0 |
| Cardiac rhythm or conduction disturbances |  | 7,014 | 26,6 | 55,193 | 26,8 |
| Heart failure |  | 3,072 | 11,7 | 22,068 | 10,7 |
| Valvular diseases |  | 2,068 | 7,9 | 15,163 | 7,4 |
| Stroke |  | 2,564 | 9,7 | 16,080 | 7,8 |
| **Respiratory diseases** |  |  |  |  |  |
| Chronic respiratory diseases (excluding cystic fibrosis) |  | 5,462 | 20,7 | 41,788 | 20,3 |
| Cystic fibrosis |  | 23 | 0,1 | 58 | 0,0 |
| Pulmonary embolism |  | 439 | 1,7 | 3,082 | 1,5 |
| **Cancer** |  |  |  |  |  |
| Female breast cancer (active) |  | 229 | 0,9 | 2,201 | 1,1 |
| Female breast cancer (under surveillance) |  | 452 | 1,7 | 4,197 | 2,0 |
| Colorectal cancer (active) |  | 245 | 0,9 | 2,257 | 1,1 |
| Colorectal cancer (under surveillance) |  | 442 | 1,7 | 4,195 | 2,0 |
| Lung cancer (active) |  | 283 | 1,1 | 1,987 | 1,0 |
| Lung cancer (under surveillance) |  | 165 | 0,6 | 1,375 | 0,7 |
| Prostate cancer (active) |  | 445 | 1,7 | 3,250 | 1,6 |
| Prostate cancer (under surveillance) |  | 703 | 2,7 | 5,852 | 2,8 |
| Other cancers (active) |  | 2,114 | 8,0 | 18,728 | 9,1 |
| Other cancers (under surveillance) |  | 1,721 | 6,5 | 13,612 | 6,6 |
| **Inflammatory and skin diseases** |  |  |  |  |  |
| Chronic inflammatory bowel diseases |  | 198 | 0,8 | 1,552 | 0,8 |
| Rheumatoid arthritis and related diseases |  | 553 | 2,1 | 4,912 | 2,4 |
| Ankylosing spondylitis and related diseases |  | 205 | 0,8 | 2,048 | 1,0 |
| Psoriasis |  | 422 | 1,6 | 3,364 | 1,6 |
| **Psychological and neurodegenerative diseases** |  |  |  |  |  |
| Neurotic and Mood Disorders, use of antidepressant treatments |  | 6,580 | 25,0 | 45,295 | 22,0 |
| Psychotics disorders, use of neuroleptics treatments |  | 1,637 | 6,2 | 11,503 | 5,6 |
| Psychiatric disorders starting in childhood |  | 25 | 0,1 | 199 | 0,1 |
| Down syndrome |  | 30 | 0,1 | 179 | 0,1 |
| Epilepsy |  | 535 | 2,0 | 3,036 | 1,5 |
| Multiple sclerosis |  | 234 | 0,9 | 1,037 | 0,5 |
| Paraplegia |  | 241 | 0,9 | 992 | 0,5 |
| Myopathy or myasthenia gravis |  | 109 | 0,4 | 631 | 0,3 |
| Parkinson disease |  | 1,162 | 4,4 | 5,752 | 2,8 |
| Dementias (including Alzheimer's disease) |  | 2,590 | 9,8 | 18,406 | 8,9 |
| Mental impairment |  | 129 | 0,5 | 1,090 | 0,5 |
| **Other pathologies** |  |  |  |  |  |
| Haemophilia or severe haemostasis disorders |  | 59 | 0,2 | 539 | 0,3 |
| HIV infection |  | 64 | 0,2 | 449 | 0,2 |
| Liver diseases |  | 682 | 2,6 | 5,010 | 2,4 |
| Chronic dialysis |  | 363 | 1,4 | 2,176 | 1,1 |
| Renal transplant |  | 414 | 1,6 | 1,825 | 0,9 |
| Cardiac transplant |  | 24 | 0,1 | 52 | 0,0 |
| Liver transplant |  | 26 | 0,1 | 141 | 0,1 |
| Lung transplant |  | 40 | 0,2 | 72 | 0,0 |

**Table S5:** Effectiveness of hybrid immunity (i.e. the joint efficacy of the second booster dose and a recent infection) against the risk of hospitalisation for COVID-19.

|  | **Case** | **%** | **Controls** | **%** | **Crude association** | **Multivariable model*** |
| --- | --- | --- | --- | --- | --- | --- |
| No recent infection and a first booster dose | 20 063 | 76% | 123 680 | 60% | 1 | 1 |
| Recent infection and a first booster dose | 807 | 3% | 20 617 | 10% | 77% (75% ; 78%) | 77% (76% ; 79%) |
| No recent infection and a second booster dose | 5 319 | 20% | 57 654 | 28% | 44% (42% ; 46%) | 45% (43% ; 47%) |
| Recent infection and second booster dose | 143 | 1% | 4 174 | 2% | 81% (77% ; 84%) | 82% (78% ; 84%) |

***** adjustment for covariates risk factors for hospitalisation for COVID-19, i.e. social deprivation index, tobacco dependence, use of immunosuppressants or oral corticosteroids, history of diabetes, dyslipidaemia, hypertension, chronic respiratory disease, other cancers (occurring before the index date), and history of SARS-COV-2 infection prior to the first booster dose.

**Table S6:** Characteristics of hospitalized cases and their controls with a first booster dose or a complete primary vaccination.

|  |  | **Case** | **%** | **Control** | **%** |
| --- | --- | --- | --- | --- | --- |
| **Sociodemographic characteristics** |  | 25,005 |  | 166,115 |  |
| **Age (year) - mean (std)** |  | 74.71 (17.2) |  | 73 (17.5) |  |
| **Age** | 12-34 | 1,152 | 4,6 | 8,623 | 5,2 |
|  | 35-44 | 688 | 2,8 | 5,100 | 3,1 |
|  | 45-54 | 1,307 | 5,2 | 10,248 | 6,2 |
|  | 55-64 | 2,204 | 8,8 | 17,028 | 10,3 |
|  | 65-69 | 1,631 | 6,5 | 11,808 | 7,1 |
|  | 70-74 | 2,722 | 10,9 | 19,693 | 11,9 |
|  | 75-79 | 3,213 | 12,9 | 22,425 | 13,5 |
|  | 80-84 | 3,538 | 14,2 | 21,639 | 13,0 |
|  | 85-89 | 4,372 | 17,5 | 26,295 | 15,8 |
|  | 90+ | 4,178 | 16,7 | 23,256 | 14,0 |
| **Sex** | Female | 12,083 | 48,3 | 80,539 | 48,5 |
|  | Male | 12,922 | 51,7 | 85,576 | 51,5 |
| **Region** | Auvergne-Rhône Alpes | 2,982 | 11,9 | 19,653 | 11,8 |
|  | Bourgogne Franche Comté | 1,231 | 4,9 | 8,251 | 5,0 |
|  | Bretagne | 1,332 | 5,3 | 9,053 | 5,5 |
|  | Centre-Val de Loire | 934 | 3,7 | 6,109 | 3,7 |
|  | Corse | 122 | 0,5 | 891 | 0,5 |
|  | DOM | 565 | 2,3 | 3,004 | 1,8 |
|  | Grand Est | 2,132 | 8,5 | 14,185 | 8,5 |
|  | Hauts de France | 2,522 | 10,1 | 17,346 | 10,4 |
|  | Ile de France | 3,527 | 14,1 | 22,809 | 13,7 |
|  | Normandie | 1,418 | 5,7 | 9,872 | 5,9 |
|  | Nouvelle Aquitaine | 2,424 | 9,7 | 16,218 | 9,8 |
|  | Occitanie | 2,221 | 8,9 | 14,869 | 9,0 |
|  | Pays de Loire | 1,199 | 4,8 | 7,975 | 4,8 |
|  | Provence Alpes Cote | 2,396 | 9,6 | 15,880 | 9,6 |
| **Social deprivation index (quintiles)** | 1 (the least deprivation) | 3,786 | 15,1 | 27,003 | 16,3 |
|  | 2 | 4,166 | 16,7 | 27,525 | 16,6 |
|  | 3 | 4,940 | 19,8 | 30,296 | 18,2 |
|  | 4 | 5,123 | 20,5 | 32,059 | 19,3 |
|  | 5 (the most deprivation) | 5,927 | 23,7 | 43,056 | 25,9 |
|  | Unknown | 1,063 | 4,3 | 6,176 | 3,7 |
| **Lifestyle habits** |  |  |  |  |  |
| Smoking |  | 2,135 | 8,5 | 10,320 | 6,2 |
| Alcoholism |  | 662 | 2,7 | 3,686 | 2,2 |
| Opioid addiction |  | 109 | 0,4 | 261 | 0,2 |
| **Vaccination schedule at the index date** |  |  |  |  |  |
|  | 2 injections | 4,166 | 16,7 | 26,439 | 15,9 |
|  | 3 injections | 20,839 | 83,3 | 139,676 | 84,1 |
| **History of SARS-COV-2 infection** |  |  |  |  |  |
| Positive PCR and antigenic tests (excluding positive tests less than 2 months before the index date ) | | 1,855 | 7,4 | 35,481 | 21,4 |
| Hospitalisation for COVID-19 (excluding hospitalisations less than 3 months before the index date ) | | 622 | 2,5 | 4,553 | 2,7 |
| **Immunosuppressive treatments** |  |  |  |  |  |
| Immunosuppressant |  | 891 | 3,6 | 6,815 | 4,1 |
| Oral corticosteroids |  | 1,428 | 5,7 | 7,916 | 4,8 |
| **Comorbidities** |  |  |  |  |  |
| **Cardiometabolic diseases** |  |  |  |  |  |
| Diabetes non insulino-treated |  | 4,085 | 16,3 | 30,117 | 18,1 |
| Diabetes insulino-treated |  | 2,022 | 8,1 | 12,085 | 7,3 |
| Obesity |  | 226 | 0,9 | 2,224 | 1,3 |
| Dyslipidaemia and lipid-lowering treatments |  | 9,030 | 36,1 | 56,074 | 33,8 |
| Hereditary metabolic diseases or amyloidosis |  | 133 | 0,5 | 777 | 0,5 |
| Hypertension |  | 16,700 | 66,8 | 106,485 | 64,1 |
| Coronary diseases |  | 4,116 | 16,5 | 24,909 | 15,0 |
| Obliterating arterial disease of the lower limb |  | 1,625 | 6,5 | 8,453 | 5,1 |
| Cardiac rhythm or conduction disturbances |  | 6,231 | 24,9 | 36,988 | 22,3 |
| Heart failure |  | 2,816 | 11,3 | 15,483 | 9,3 |
| Valvular diseases |  | 1,784 | 7,1 | 10,193 | 6,1 |
| Stroke |  | 2,340 | 9,4 | 11,227 | 6,8 |
| **Respiratory diseases** |  |  |  |  |  |
| Chronic respiratory diseases (excluding cystic fibrosis) |  | 5,128 | 20,5 | 32,493 | 19,6 |
| Cystic fibrosis |  | 24 | 0,1 | 48 | 0,0 |
| Pulmonary embolism |  | 398 | 1,6 | 2,209 | 1,3 |
| **Cancer** |  |  |  |  |  |
| Female breast cancer (active) |  | 229 | 0,9 | 1,768 | 1,1 |
| Female breast cancer (under surveillance) |  | 420 | 1,7 | 3,080 | 1,9 |
| Colorectal cancer (active) |  | 233 | 0,9 | 1,734 | 1,0 |
| Colorectal cancer (under surveillance) |  | 372 | 1,5 | 2,782 | 1,7 |
| Lung cancer (active) |  | 269 | 1,1 | 1,425 | 0,9 |
| Lung cancer (under surveillance) |  | 143 | 0,6 | 944 | 0,6 |
| Prostate cancer (active) |  | 392 | 1,6 | 2,069 | 1,3 |
| Prostate cancer (under surveillance) |  | 569 | 2,3 | 3,593 | 2,2 |
| Other cancers (active) |  | 1,696 | 6,8 | 12,727 | 7,7 |
| Other cancers (under surveillance) |  | 1,475 | 5,9 | 9,030 | 5,4 |
| **Inflammatory and skin diseases** |  |  |  |  |  |
| Chronic inflammatory bowel diseases |  | 187 | 0,8 | 1,323 | 0,8 |
| Rheumatoid arthritis and related diseases |  | 474 | 1,9 | 3,640 | 2,2 |
| Ankylosing spondylitis and related diseases |  | 180 | 0,7 | 1,728 | 1,0 |
| Psoriasis |  | 381 | 1,5 | 2,660 | 1,6 |
| **Psychological and neurodegenerative diseases** |  |  |  |  |  |
| Neurotic and Mood Disorders, use of antidepressant treatments |  | 5,999 | 24,0 | 34,073 | 20,5 |
| Psychotics disorders, use of neuroleptics treatments |  | 1,631 | 6,5 | 9,320 | 5,6 |
| Psychiatric disorders starting in childhood |  | 35 | 0,1 | 296 | 0,2 |
| Down syndrome |  | 31 | 0,1 | 199 | 0,1 |
| Epilepsy |  | 547 | 2,2 | 2,457 | 1,5 |
| Multiple sclerosis |  | 221 | 0,9 | 959 | 0,6 |
| Paraplegia |  | 251 | 1,0 | 847 | 0,5 |
| Myopathy or myasthenia gravis |  | 104 | 0,4 | 474 | 0,3 |
| Parkinson disease |  | 1,018 | 4,1 | 3,828 | 2,3 |
| Dementias (including Alzheimer's disease) |  | 2,314 | 9,3 | 11,550 | 7,0 |
| Mental impairment |  | 154 | 0,6 | 1,061 | 0,6 |
| **Other pathologies** |  |  |  |  |  |
| Haemophilia or severe haemostasis disorders |  | 58 | 0,2 | 393 | 0,2 |
| HIV infection |  | 72 | 0,3 | 392 | 0,2 |
| Liver diseases |  | 664 | 2,7 | 3,964 | 2,4 |
| Chronic dialysis |  | 195 | 0,8 | 1,020 | 0,6 |
| Renal transplant |  | 256 | 1,0 | 864 | 0,5 |
| Cardiac transplant |  | 8 | 0,0 | 21 | 0,0 |
| Liver transplant |  | 13 | 0,1 | 80 | 0,1 |
| Lung transplant |  | 25 | 0,1 | 25 | 0,0 |

**Table S7:** Reduced risk of hospitalization for COVID-19 in the Omicron period associated with the first booster dose compared with complete primary vaccination schedule, overall and by time since the first booster dose.

|  | **Case** | **%** | **Control** | **%** | **Crude association** | **Multivariable model*** |
| --- | --- | --- | --- | --- | --- | --- |
| Complete primary vaccination | 4,166 | 17% | 26,439 | 16% | 1 | 1 |
| First booster dose | 20,839 | 83% | 139,676 | 84% | 7% (4% ; 11%) | 25% (22% ; 28%) |
| Complete primary vaccination | 4,166 | 17% | 26,439 | 16% | 1 | 1 |
| First booster dose: 14 days to < 2 months | 125 | 0% | 2,048 | 1% | 64% (57% ; 70%) | 64% (57% ; 71%) |
| First booster dose: 2 to < 4 months | 394 | 2% | 3,732 | 2% | 37% (29% ; 43%) | 41% (34% ; 47%) |
| First booster dose: ≥ 4 months | 20,320 | 81% | 133,896 | 81% | 5% (2% ; 9%) | 24% (21% ; 27%) |

***** adjustment for social deprivation index, tobacco dependence, use of immunosuppressants or oral corticosteroids, history of diabetes, dyslipidaemia, hypertension, chronic respiratory disease, other cancers (occurring before the index date), and history of SARS-COV-2 infection prior to the complete primary vaccination.
